# Limbal niche cells are a potent resource of adult mesenchymal progenitors

**DOI:** 10.1111/jcmm.13635

**Published:** 2018-04-20

**Authors:** Ping Guo, Hong Sun, Yuan Zhang, Sean Tighe, Shuangling Chen, Chen‐Wei Su, Yongsong Liu, Hongxia Zhao, Min Hu, Yingting Zhu

**Affiliations:** ^1^ Shenzhen Eye Hospital Shenzhen Key Laboratory of Department of Ophthalmology School of Optometry & Ophthalmology Shenzhen University Shenzhen China; ^2^ Department of Ophthalmology the First Affiliated Hospital of Nanjing Medical University Nanjing China; ^3^ R and D Department Tissue Tech, Inc. Miami FL USA; ^4^ Department of Ophthalmology Yan’ An Hospital of Kunming Kunming China; ^5^ Department of Ophthalmology the Second People's Hospital of Yunnan Province Kunming China

**Keywords:** cell‐based therapy, differentiation, limbal stem cell deficiency, limbus, niche, stem cell

## Abstract

Limbal niche cells located in the limbal Palisades of Vogt are mesenchymal stem cells that reside next to limbal basal epithelial cells. Limbal niche cells are progenitors that express embryonic stem cell markers such as Nanog, Nestin, Oct4, Rex1, Sox2 and SSEA4, mesenchymal cell markers such as CD73, CD90 and CD105, and angiogenesis markers such as Flk‐1, CD31, CD34, VWF, PDGFRβ and α‐SMA, but negative for CD45. In addition, the stemness of limbal niche cells can be maintained during their cell culture in a three‐dimension environment. Furthermore, expanded limbal niche cells have the capability to undergo adipogenesis, chondrogenesis, osteogenesis and endogenesis in vitro, indicating that they are in fact a valuable resource of adult progenitors. Furthermore studies on how the limbal niche cells regulate the aforementioned stemness and corneal fate decision are warranted, as those investigations will shed new light on how mesenchymal progenitors reverse limbal stem cell deficiency and lead to new methods for limbal niche cell treatment.

## 1 HUMAN LIMBUS

Anatomically the limbal stem cell (SC) niche is located in the region termed Palisades of Vogt. Unlike the structure in the cornea, the basement membrane from the limbus is interconnecting with the stroma,^1^ and the limbal basal epithelial cells have invaginations through the basement membrane to connect with the underlying matrix,^2^ suggesting that limbal epithelial SC keep close contact with the limbal stroma cells. Limbal SC extends into the limbal stroma, forming a structure called “limbal epithelial crypt” (LEC).^1^ Interestingly, limbal crypts are encircled by focal stromal projections (FSP), circulating down the projections of the limbal epithelium closely linked to the limbal vasculature.^3^ Such limbal epithelial progenitor cells (LEPC) retain their progenitor status in the limbal niche environment (reviewed in 4). When malfunctions among limbal niche cells (LNC) and LEPC occur, it causes a state of limbal stem cell deficiency (LSCD), leading to symptoms such as redness, irritation, photophobia, decreased vision from corneal opacity and irregular astigmatism (reviewed in 5). The aetiology for LSCD remains unclear.

## 2 HETEROGENEITY OF LIMBAL STEM CELLS

Previous studies indicate that limbal SC are distributed in the limbal basal layer heterogeneously, suggesting a heterogeneously localization of the limbal niche. The limbal basal epithelial cells heterogeneously express Vim,^6^ ΔNp63α,^7^ ABCG2,^8^ C/EBPδ and Bmi‐1,^9^ while homogeneously expressing CK15 that extends to conjunctival basal epithelial cells.^10^ N‐cadherin can also be expressed by putative stem cells or progenitors as well as melanocytes in the human limbal epithelial SC niche.^11^ Cx43, on the other hand, is well demonstrated to be a negative marker of limbal SC, but found positive in epithelial cells in LEC.^1^ However, it remains unclear how the aforementioned SC and molecules modulate the quiescent and/or active stage of limbal epithelial SC.

## 3 ISOLATION AND EXPANSION OF HUMAN LIMBAL NICHE CELLS

The limbal stem cell niche is a structure of the ocular surface that is characterized by high specification, organization and clinical significance (Reviewed in 12). Limbal stromal niche cells expressing SC markers can be isolated and expanded without differentiation and maintain clonal growth of limbal epithelial progenitors.^13^ Culture of such cells on denuded amniotic membrane can maintain native niche cells^14^ and quiescence.^15^ Several types of cells enriched in the limbal niche may serve as niche cells, including melanocytes,^11^ blood vascular endothelial cells^3^ and neural cells.^16^ Although the presence of niche cells (NC) is implicated by the aforementioned studies, it has not been identified until recently when a novel isolation method using collagenase A was used.^17^ This method helps to identify putative NC as small Vim positive stromal cells in the form of cell clusters.^17^, ^18^ In addition, expansion of the niche/stem cell population isolated from collagenase A digestion is more efficient than single cell suspension by trypsin/EDTA digestion based on the number of p63α positive cells,^19^ suggesting that an intact cell‐cell contact help to maintain LSC in an undifferentiated state in vitro during expansion. Compared to the method of dispase digestion which removes intact sheets of limbal epithelium 20 but not all limbal epithelial progenitors, collagenase A digestion removes the entire progenitors in the limbal basal area which express ESC markers such as Nanog, Oct4 and Sox2.^18^ In addition, collagenase A digestion has been performed without shaking, as shaking is not as effective in the concept of releasing progenitor cells.^21^

Modified embryonic stem cell medium (MESCM) is considered an optimal medium for expansion of limbal niche cells. MESCM is comprised of DMEM/F‐12 (1:1) but supplemented with 10% knockout serum, 5 μg/mL insulin, 5 μg/mL transferrin, 5 ng/mL sodium selenite, 4 ng/mL bFGF and 10 ng/mL hLIF, which is similar to another widely used medium containing DMEM‐F12, knockout serum, basic fibroblast growth factor and leukaemia inhibitory factor (LIF) which produces a cell phenotype closest to that of a pluripotent stem cells.^22^ In fact, such stem cells in the limbal niche have been confirmed (for review, see 23 and 24). Maintenance of close association between the epithelial and niche cells lead to clonal growth in a low‐calcium, serum‐free medium while disruption of this linkage with trypsin/EDTA abolishes clone growth unless cocultured with 3T3 feeder layers.^17^ Interestingly, Bhartiya et al^25^ have isolated a group of stem cells, named very small embryonic‐like stem cells (VSEL). The phenotype of such potent cells includes a very small size (3‐5 μm), high nucleo‐cytoplasmic ratio, and expression of Oct‐4, SSEA‐4, and other pluripotent markers such as Nanog, Sox‐2, Rex‐1 and Tert.^25^ Similarly, on epithelial‐denuded amniotic membrane, maintenance of such linkage may cause extensive epithelial outgrowth, which can be attenuated by trypsin/EDTA treatment.^17^ Epithelial outgrowth from the clusters obtained from collagenase digestion may be significantly larger. In addition, single cells obtained from clonal growth may generate more holoclones when cultured on 3T3 fibroblast feeder layers than those obtained from dispase digestion,^17^ suggesting that these cells are indeed young progenitors. Interestingly, human corneal stromal progenitors exhibit survival capacity following isolation from a stored organ.^26^ Furthermore, mesenchymal stromal progenitors cultivated from the limbus display immunosuppressive qualities in addition to their established non‐immunogenic profile and stimulate limbal epithelial cell growth in vitro across species boundaries.^27^ Using a novel method of serial passages on a 2D Matrigel system, we further isolated and expanded LNC to up to 12 passages with 33 population doublings and characterized them as angiogenesis progenitors when cultured in embryonic stem cell medium (ESCM) with fibroblast growth factor‐basic (bFGF) and LIF.^28^ The cells are thought to be angiogenesis progenitors due of up‐regulation of these specific progenitor markers when the cells are reseeded in 3D Matrigel.^28^ These cells may be differentiated into vascular endothelial cells along with pericytes for stabilization of tube network generated by human umbilical vein endothelial cells (HUVEC).^28^ Interestingly, stromal cells which express a number of angiogenesis markers are located perivascularly, next to epithelial cells in the limbal basal area.^29^ If cultured in a 3D Matrigel system, the stromal cells obtained by dispase/collagenase digestion but not the stromal cells at the limbal residual area may form the spheres with angiogenesis progenitors to maintain vascular networks.^29^ Such sphere‐forming cells from peripheral cornea demonstrate the ability to repopulate the ocular surface, expressing stem cell markers such as ΔNp63α, ABCG2, ABCB5 as well as the basal limbal and putative niche marker notch 1.^30^ Similar to the cells obtained by collagenase digestion, the cells obtained from dispase/collagenase digestion may also proliferate when cultured on 2D Matrigel for 12 passages. As a result, the spindle cells may express the specific markers of MSC and angiogenesis, have higher ability to form colonies, and retain higher efficiency of tri‐lineage differentiation, when compared to the stromal cells isolated by dispase/collagenase digestion from the limbal residual area when cultured in DMEM containing 10% fetal bovine serum (FBS).^29^ Although limbal NC or HUVEC may reunite with LEPC to generate the spheres with higher expression of CK15, C/EBPδ and ΔNp63α, limbal NC but not HUVEC can attenuate over‐expression of CK12 keratin in LEPC.^28^ Those findings raise the hypothesis that a vascular niche also exist in the human limbal niche. The above findings also suggest that we may devise another novel method of separating limbal basal epithelial cells into two compartments, that is, one removed solely by dispase and the other that dispase cannot remove. The latter can be obtained by collagenase A digestion.

## 4 HUMAN LIMBAL NICHE CELLS AS ANGIOGENESIS PROGENITORS

### 4.1 Vascular/SC niche

The vascular niche refers to a microenvironment that is generated by vascular endothelial cells (VEC), pericytes and bone marrow (BM) cells.^31^ The unique perivascular localization of the central neural system, the bone marrow and the testis suggests that vascular niche plays a critical role within the SC niche.^32^, ^33^, ^34^ Vascular endothelium can also support bone marrow SC in the central nervous system, the muscle and the pancreatic islets.^35^ The mechanisms involved include the physical contact and the paracrine factors such as bFGF, IGFBP2, angiopoietin‐1 (Ang1), BMP4, DHH, PEDF, NDEF, FGF4 and SDF‐1.^32^ VEC are derived from their endothelial progenitor cells (EPC). Vascular BM, composed of collagen IV, laminin, nidogens, heparin sulfate proteoglycan and growth factors such as FGF, TGFβ, IGF, PDGF, EGF are a key part of all vascular niches. Adult tissue needs a highly selective extracellular matrix (ECM) to maintain their SC properties particularly during development.^33^ For example, human ESC may retain their undifferentiated state when cultured on Matrigel.^34^ BM can also improve the proliferation and differentiation of human MSC in vitro.^36^ For instance, collagen IV may promote the differentiation potential of ESC to EPC^37^ whereas laminin can promote insulin gene expression and proliferation in beta islet cells, and beta1‐integrin is required for the beta islet cell response to laminin's signals from VEC.^38^ Hematopoietic stem cells (HSC) may shift from a quiescent osteoblastic niche to a vascular niche to support their proliferation and further differentiation in the bone marrow.^32^ Previously, we have isolated and expanded a group of limbal niche cells on 2D Matrigel in MESCM. We attribute the success of the isolation and expansion of LNC to 2D Matrigel culture, as the expanded cells are in contact with the basement membrane continuously.

### 4.2 Pericytes, smooth muscle cells and vascular niche

There are two kinds of mural vessel cells (pericytes and smooth muscle cells [SMC]) which share a similar function of controlling the vessel contraction and new blood vessel generation. Pericytes make specific cell‐to‐cell contacts with VEC of capillaries, postcapillary venues, precapillary arterioles, and collecting venules, while SMC are distributed in the middle layer of bigger vessel walls. In fact, pericytes are an important compartment of the vascular niche because of their contribution to MSC^39^ and ability to produce BM.^40^ Pericytes may also be recruited during formation of vasculogenic tube assembly in order to stimulate endothelial BM formation, and are enveloped by BM, indicating the essential requirement of BM for the pericyte niche.^41^ Pericytes also give rise to astrocytes and deposit ECM in the injured spinal cord.^42^ Central neural system (CNS) pericytes can differentiate into macrophage/dendritic cells, antigen presenting cells, and MSC.^43^ Nevertheless, it is unclear whether pericytes may serve as limbal NC.

Multi‐potential MSC derived from human tissues, including adipose tissue and skeletal muscle, appear to be derived nearly exclusively from perivascular SMC‐pericytes.^44^, ^45^, ^46^ A variety of contractile proteins are important for the differentiated function of the SMC, including α‐SMA, smooth muscle (SM) myosin heavy chains (MHC), SM myosin light chains, h1‐calponin and SM α‐tropomyosin. In addition, differentiated SMC express a number of proteins that are part of the cytoskeleton and/or are involved in the regulation of contraction molecules such as h‐calponin, SM22α, h‐caldesmon, β‐vinculin, metavinculin, telokin, smoothelin, LPP and desmin. Interestingly, virtually all of these SMC differentiation markers can also be expressed in non‐SMC under some conditions, with the possible exception of the SM MHC isoforms,^47^ which appear to be the most specific markers of differentiated SMC.^45^

Pericytes represent a unique subtype of perivascular cells with multi‐lineage developmental features and various angiogenic functions.^48^ As the ancestor of MSC, pericytes can not only give rise to MSC, but also contribute indirectly to tissue regeneration, possibly by promotion of angiogenesis and elimination of inflammation of endogenous progenitor cells. Therefore, pericytes could be applied as alternative therapeutic cells in replacement of MSC for regenerative medicine.^49^ In order to select the right cell type, there are clues for the relation between the marker pattern and the multipotent potential of such cells. For example, PDGFRβ‐expressing cells are perivascular cells distinct from mature pericytes due to their ability to differentiate into mature pericytes and to support vascular tube stability and survival in vitro.^50^ Most pericytes in vivo are α‐SMA negative 51 except those located near arterioles are routinely α‐SMA positive.^52^ Interestingly, less than 5% of freshly isolated capillary pericytes express α‐SMA, but nearly 100% express this marker in 7 days in culture with serum.^53^ Human and rat brain pericytes are positive for stem cell markers such as NG2 and nestin.^43^ CD146 can be used in isolating pericytes expressing PDGFR‐β, α‐SMA and NG2 from human skeletal muscle, pancreases, placenta, heart, skin, lung, brain, eye, gut, bone marrow and umbilical cord.^44^ Although some markers are commonly used for progenitor cell identification, such as α‐SMA, PDGFR‐β, NG2, RGS5, aminopeptidase A and N5‐7, those markers are not universal, but rather specific to the developmental stage, tissue bed, and even species.^54^ ACTA2, SMAA, SM22, MYOCD, MYH11, SMMHC and CNN1 could be used to identify the presence of SMC.^46^ The in vitro data support the concept that in the correct environment, CNS pericytes may differentiate to MSC and then differentiate to bone, adipocytes, smooth muscle, and VEC when cultured in a medium containing 10% FBS and 50 ng/mL vascular endothelial growth factor (VEGF).^52^ ESC could turn into Flk‐1 positive vascular progenitors and further differentiate into VEC in the presence of VEGF or SMC and PDGFβ.^37^

### 4.3 Endothelial progenitor cells and the stem cell niche

Endothelial progenitor cells (EPC) can be found and replicated from human limbus for tissue engineering purposes.^55^, ^56^ EPC is also an important angiogenesis progenitor cells identified to be positive for Flk‐1, CD34, CD31, CD73, CD90, CD105, CD144 and negative for CD10, CD13, CD29, CD44. CD31 is the key marker of EPC and mature VEC, identified as a regulator of adhesion, migration and activation.^57^ Flk1 is the earliest marker of angioblast precursors, specifically for a subset of cells that migrate into the extraembryonic yolk sac to form the vascular plexus during the murine development.^58^ Foetal mouse lung mesenchymal cells with the highest Flk‐1 expression can be differentiated into endothelium more efficiently.^59^ EPC could be isolated from bone marrow, blood^60^ and non‐BM adult tissues, including skeletal muscle, adipose, spleen, liver, intestine and myocardium.^61^ In the bone marrow, EPC derive from bone marrow stem cells and exist as residential progenitor cells. Such stem cells in limbal stroma are in fact multi‐functional niche cells^23^ and interact with limbal epithelial cells to exert their functions.^24^, ^62^ Limbal ESC obtained from young donors may produce better stem cells for clinical therapies.^63^ Sphere forming cells are also a promising cell type for stem cell repopulations 30, 64 and native LNC may promote the expansion of LEPC.^19^ Human corneal stromal SC may survive in stored organ or culture,^26^ although biomechanics exhibit a powerful effect on LNC and their growth and differentiation.^12^ Human corneal stromal SC may prevent corneal scarring.^65^ Stem cells and niche cells may interact with cell adhesive molecules to exert their functions.^66^ Corneal stromal SC can even be induced to functional corneal endothelium by activation of Wnt signalling.^67^ Human corneal stromal SC can also support limbal epithelial cells in vitro.^68^ However, it is unclear whether EPC could serve as SC niche cells or serve as limbal niche cells.

## 5 HUMAN LIMBAL NICHE CELLS AS A SOURCE OF MESENCHYMAL PROGENITORS

MSC clusters represent an important component of the limbal‐niche.^29^ MSC have great potential for regenerative medicine due to their high plasticity, self‐renewal, specific immune response and the ability for genetic modification. Therefore, the growing demand for cell‐based therapy necessitates a massive production of MSC. According to the minimum criteria set by the International Society for Cellular Therapy, MSC should be (i) adhere to plastic, (ii) express of specific surface antigens such as CD73, CD90 and CD105 positive, CD45 and CD34 negative, and (iii) have multi‐lineage differentiation potential, for example, to adipocyte, chondrocytes and osteocyte.^69^ Although first isolated from the bone marrow,^70^ MSC could virtually be obtained from all adult tissues.^39^, ^71^ MSC are located in perivascular sites of many tissues such as in the bone marrow,^43^ in adipose tissue, placenta and skeletal muscle^44^ and in the dental pulp.^72^ Functional corneal endothelial cells could be derived from corneal stroma stem cells of neural crest origin by retinoic acid and activation of Wnt/β‐Catenin Signalling.^67^ MSC could also be derived from the human limbal niche.^28^, ^29^

The cell origin of the MSC, that is, the progenitor cells of MSC, has not been very well‐defined. However, recent evidence indicates that the progenitor cells of MSC lie in a parivascular niche and that MSC derive from the pericytes, or perivascular progenitor cells.^39^, ^44^ Pericytes and MSC express the cell markers in their native state, for example, α‐SMA, CD44, CD73, CD90 and CD105, NG2, PDGFR‐β and stromal progenitor antigen‐1 (STRO‐1) but not CD45, CD31.^44^, ^72^, ^73^, ^74^, ^75^ In in vitro culture, pericytes display an MSC‐like phenotype, with respect to morphology, clonal growth, self‐renewal and tri‐lineage differentiation to different tissues such as bone, cartilage, fat,^44^ nervous tissue^43^ and skeletal muscle.^73^, ^76^ Meanwhile, MSC could be induced into EPC by VEGF, expressing Flk‐1, CD34 and CD31.^77^ Human bone marrow derived MSC express CD31, α‐SMA and smoothelin upon interaction with endothelial cell matrix.^78^ Under serum‐deprived conditions, human bone marrow derived MSC express prosurvival and angiogenic factors including VEGF‐A, ANGPTs, IGF‐1 and HGF, and a population of these cells have the potential to differentiate into endothelial like cells.^79^ Multipotent foetal mouse lung mesenchymal cells with the highest Flk‐1 expression may be differentiated into endothelia more efficiently.^59^

MSC could also be isolated from the niche of epithelial stem cells^80^ or differentiated from embryonic stem cells,^54^ while pericytes, which is believed to be the origin of MSC, could also be differentiated from human pluripotent SC.^48^ Sox2 positive skin‐derived precursors, which localize in the niche of the hair papillae and whisker follicles, represent an endogenous embryonic precursor cells that exhibit multipotency properties similar to embryonic neural‐crest stem cells^80^ and can be easily differentiated into functional vascular smooth muscle cells (SMC) in a serum‐free condition. Based on the marker expression pattern of isolated MSC, a group of stem cell markers including Oct4, PDGFRβ and SSEA4 may be detected in the primary but not expanded MSC,^81^ indicating that Oct4, PDGFRβ and SSEA4 may be used as the specific markers of MSC. However, different markers used in isolating MSC from different types of tissue are problematic for identification and comparison of the similarity and differences reported from various authors.

It has been well documented that MSC within the niche of hematopoietic stem cells is a critical component of the SC niche. MSC contribute to the complex structure of the hematopoietic niche by differentiating into osteoblasts and by supporting HSC proliferation and self‐renewal by themselves. MSC also participate in the hematopoietic process through releasing paracrine signalling factors.^82^ Both pericytes and MSC can provide mechanical and paracrine support to other cell types.^83^ It is unclear whether the limbal niche derived MSC could serve as niche cells for the epithelial SC. Another notion noticed is that MSC may be tissue specific, thus we may speculate that LNC, which is progenitor cells of both MSC and angiogenesis progenitor cells, may act as the best support cells than other tissue derived MSC.

## 6 GROWTH FACTORS AND OTHER ACTIVE LIGANDS AFFECTING BEHAVIOUR OF LIMBAL NICHE CELLS

Growth factors such as bFGF and TGF‐β3 may induce multilayered lamellae with orthogonally oriented collagen fibrils and the human corneal stromal tissue. The approach of combining the effect of growth factors and substrates provides a new regenerative approach for corneal regeneration and medical treatment.^84^ PDGF and VEGF can affect cell differentiation from ESC. Flk‐1 positive progenitor cells from ESC may be induced into VEC by VEGF.^37^ Pericytes isolated from CNS may differentiate to MSC and then differentiate to bone, adipocytes, smooth muscle and VEC with 10% fetal calf serum and 50 ng/mL VEGF.^52^ The influence of VEGF and PDGF on the outcome of angiogenesis progenitor expansion from the human limbus is not clear. BMP4 synergizes with LIF to maintain self‐renewal by activating JAK/Stat3 signalling in mouse ES cells in the presence of serum.^85^ In the absence of serum, BMP synergizes with LIF to maintain self‐renewal in mouse ES cells through induction of gene expression of ID genes.^86^ In an in vitro reunion model of LEPC and LNC in 3D Matrigel, LEPC+LNC spheres shows their ability of significantly higher clonal growth and dramatic lower differentiation in corneal epithelium.^87^ In such a culture, nuclear translocation of pSmad1/5/8 is observed in LEPC, but not in LNC. Such a nuclear translocation of pSmad1/5/8 is associated with activation of canonical BMP signalling.^87^ In addition, β‐catenin was stabilized in the perinuclear cytoplasm in LEPC and correlated with up‐regulation of Wnt7A and FZD5 preferentially expressed by LEPC.^87^ Interestingly, addition of BMP inhibitor noggin inhibits canonical BMP signalling but activates canonical Wnt signalling, indicating balancing action of canonical Wnt signalling and canonical BMP signalling between LNC and LEPC mediates clonal growth of LEPC.^87^ It is unclear whether canonical BMP signalling is also involved in induction of ESC like cells in the human limbus into angiogenesis progenitors.

## 7 ANIMAL MODELS TO STUDY DEFICIENCY OF LIMBAL NICHE CELL DYSFUNCTION

Recently, a mice model was established to study the mechanisms of limbal stem cell deficiency (LSCD), suggesting that the phenotype of LSCD in the mouse model was maintained for more than 3 months.^88^ A rabbit model was also established for potential use of limbal MSC to treat corneal disorders.^27^

## 8 SUMMARY

**Figure 1:**
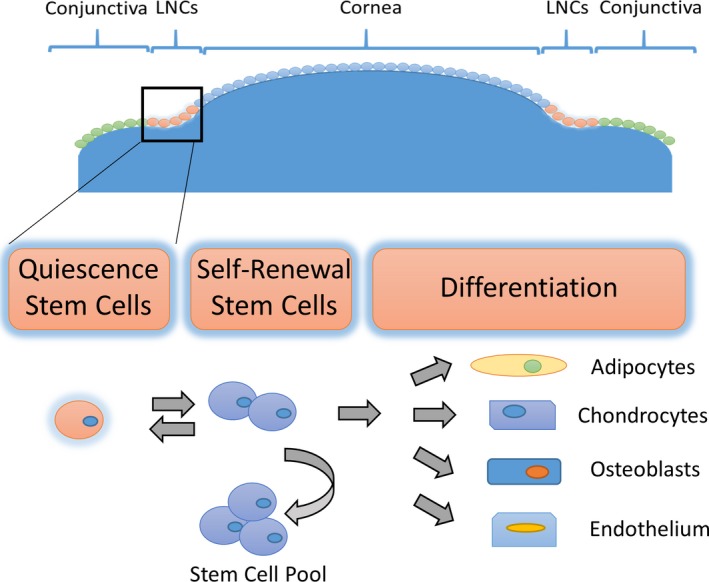
Summary of Limbal Niche Cell Function. Limbal niche cells are an important source of mesenchymal stem cells located in the limbal palisades of vogt, next to limbal basal epithelial cells. The stemness of limbal niche cells could be maintained during their culture in a three‐dimension environment. The expanded limbal niche cells have the capability to undergo adipogenesis, chondrogenesis osteogenesis and endogenesis in vitro*,* indicating that they are in fact a valuable resource of adult progenitors

Limbal niche cells are another source of local mesenchymal stem cells located in the limbal Palisades of Vogt, next to limbal basal epithelial cells. The cells are as small as 5 μm in diameter. Limbal niche cells can be isolated from the human limbus by collagenase digestion and cultured in DMEM plus 10% FBS or in MESCM with bFGF and LIF on plastic, on 2D Matrigel, or on 3D Matrigel. Characteristically limbal niche cells are positive for various stem cell markers (Oct4, Sox2, Nanog, Rex1, SSEA4, Nestin and N‐cadherin), mesenchymal cell markers (CD73, CD90, and CD105), and angiogenesis markers (α‐SMA, CD31, CD34, Flk‐1, PDGFRβ and VWF), but negative for CD45. The expanded LNC are capable of differentiating into adipocytes, osteoblasts and chondrocytes in vitro. Recently, we have successfully isolated human LNC by collagenase digestion of the entire limbal tissue. We have demonstrated LNC express cell markers for BMMSC and ESC, indicating that they may be the progenitor cells for MSC. Furthermore, LSC/LEPC could be maintained during the culture process of LSC/LEPC in a three‐dimension environment. Furthermore studies on how the LNC may regulate the stemness of LEPC and the decision of corneal fate are warranted, which may shed new light on how MSC are used for treatment of diseases with stem cell deficiency, for example, managing to reverse LSCD. The primary functions of LNC are summarized as Figure 1:

## CONFLICT OF INTEREST

The authors confirm that there is no conflict of interest.
